# Occurrence of paratesticular ganglioneuroma 18 years after concurrent adrenal ganglioneuroma and papillary thyroid carcinoma – a case report

**DOI:** 10.1186/s12885-019-6440-4

**Published:** 2019-12-30

**Authors:** Chu-Wen Fang, Jyh-Seng Wang, Tony T. Wu, Jen-Tai Lin

**Affiliations:** 1 Department of Surgery, Kaohsiung Veterans General Hospital, Kaohsiung, Taiwan; 2 Division of Urology, Department of Surgery, Chi Mei Medical Center, Tainan, Taiwan; 3 Department of Pathology, Kaohsiung Veterans General Hospital, Kaohsiung, Taiwan; 4 Division of Urology, Department of Surgery, Kaohsiung Veterans General Hospital, No.386, Ta-Chung 1st Rd, 81362 Kaohsiung, Taiwan

**Keywords:** Ganglioneuroma, Papillary thyroid carcinoma (PCT), Paratesticular tumour, Multiple endocrine neoplasm (MEN), RET

## Abstract

**Background:**

Ganglioneuromas (GNs) are composed of mature ganglion cells and Schwann cells with a fibrous stroma; GNs are most often observed in children and young adults. The majority of non-cranial GNs are located in the retroperitoneum and posterior mediastinum. Other reported rare sites include the adrenal gland, small intestine, colon and urinary bladder. However, para-testicular GNs are even more rare.

**Case presentation:**

Herein, we report the case of a patient with concurrent adrenal GN and thyroid papillary carcinoma who developed paratesticular GN eighteen years later.

**Conclusions:**

We conclude that there is an association among papillary thyroid carcinoma, GN and MEN2 syndromes. This case report may provide important information for the proposed association. However, further studies are required.

## Background

Tumours originating mainly from primordial neural crest cells may include neuroblastoma, ganglioneuroblastoma, and ganglioneuroma (GN), while GN is the most benign among these tumours [1]. GNs are composed of mature ganglion cells and Schwann cells with a fibrous stroma, and they are often observed in children and young adults with a slightly female predominance. In addition, non-cranial GNs (i.e., incidentaloma) are frequently asymptomatic and detected incidentally. Most non-cranial GNs are located in the retroperitoneum and posterior mediastinum [2, 3]. However, other rare sites of non-cranial GNs may include the adrenal gland, small intestine, colon, urinary bladder and para-testis [4–7].

It has been reported that the prevalence of adrenal incidentaloma is approximately 0.2, 3, and 7% in young patients, those past their fifth decade, and those past their seventh decade, respectively [4]. The differential diagnosis of adrenal incidentalomas may vary from a simple benign cyst or lipoma to adrenal carcinoma.

Para-testicular tumours are indolent, slowly growing masses, of which 70% are benign in nature [8]. They may manifest as scrotal masses and are easily confused with testicular tumours [9]. The most commonly reported benign para-testicular tumours are lipomas, adenomatoid tumours and leiomyomas [10, 11]. Para-testicular GN has rarely been reported.

Herein, we report our experience with a patient with previous adrenal GN and concurrent thyroid papillary carcinoma, who developed paratesticular GN eighteen years later.

### Case presentation

A 60-year-old male with a history of type II diabetes mellitus and hypertension for more than 9 years visited our out-patient department (OPD) for evaluation of prostate status based on family history of prostate cancer. However, the patient denied a family history of multiple endocrine neoplasms (MENs).

Tracing back his history, it was found that he had right adrenal incidentaloma eighteen years ago when he was 42 years old. At that time, his body weight decreased by approximately 3–4 kg without other comorbidities. In addition, he denied Cushing appearance, elevated blood pressure and palpation or episodes of headache/hypertension/palpitations. The abdominal sonograph showed an 8.7 cm hypoechoic mass over the right adrenal gland, while a further MRI examination revealed a large heterogeneous mass over the right adrenal gland with blurred margins of the right adrenal gland and liver. The endocrine workup displayed normal serum aldosterone and dehydroepiandrosterone sulfate (DHEAS) levels, whereas the cortisol level was suppressed in the low dose overnight dexamethasone test; there was also an elevation of urine vanillylmandelic acid (VMA), serum carcinoembryonic antigen (CEA), alpha foetal protein (AFP) and lactate dehydrogenase (LDH) (13.6 mg/day, 5.77 ng/mL, 6.35 ng/mL, and 160 IU/L, respectively).

**Fig. 1:**
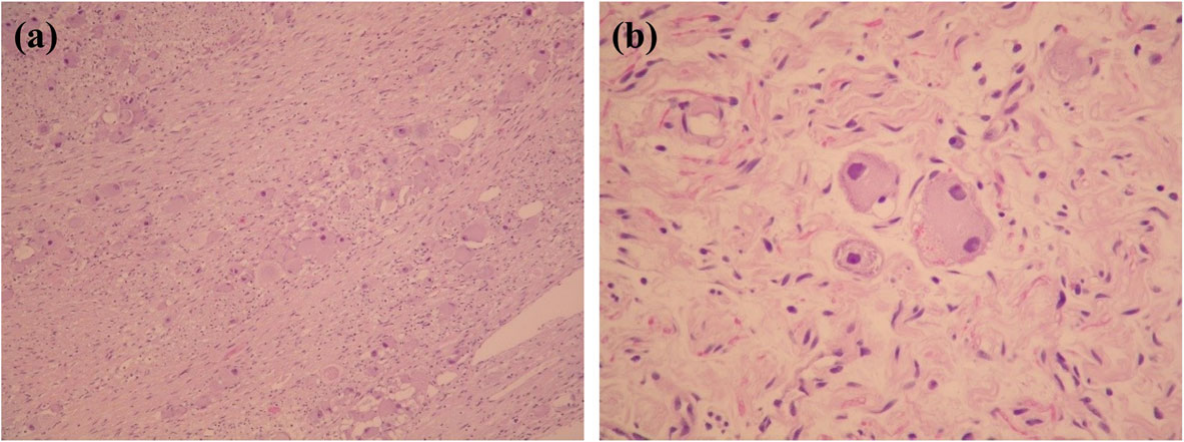
**a**. Adrenal ganglioneuroma, Schwann cells and fibrous stroma with scattered mature ganglion cells (HE, × 10). **b**. Adrenal ganglioneuroma, mature ganglion cells, one binucleated, in a background of Schwann cell fascicles (HE, × 40)

**Fig. 2:**
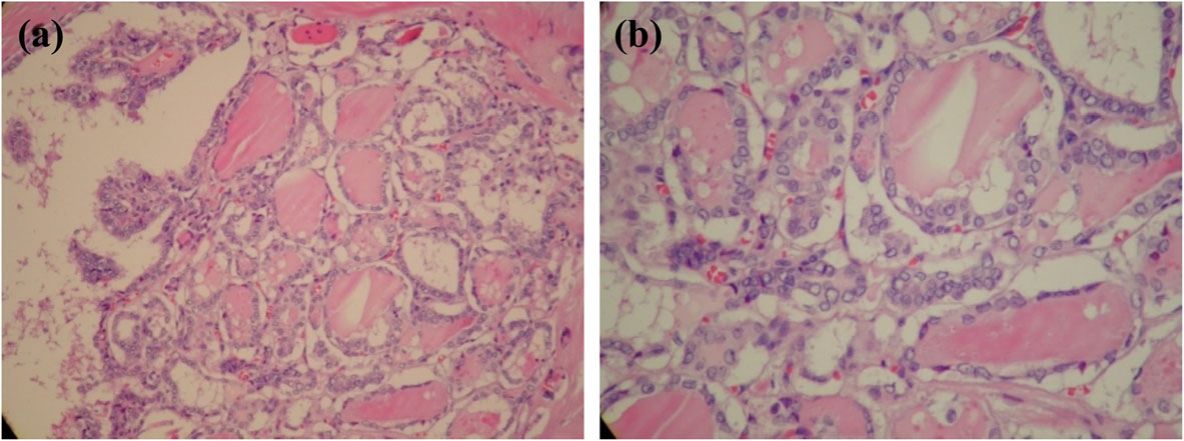
**a**. Papillary thyroid carcinoma with a follicular and focal papillary growth pattern (HE, × 20). **b**. Papillary thyroid carcinoma with characteristic nuclear clearing and grooving (HE, × 40) Infiltrative with fibrous trabeculation, psammoma bodies, strongly eosinophilic colloid with scalloping

Under the assumptions of adrenal pheochromocytoma and a potentially increased risk of malignancy, he underwent right adrenalectomy and simple nephrectomy due to intraoperative severe adhesions of the right kidney. The pathology diagnosis was a ganglioneuroma composed of abundant Schwann cells ornamented with scattered mature ganglion cells and foci of calcification. (Figs. 1-1 and 1-2).

Nonetheless, enlarged lymph nodes on the left side of neck were noted concurrently after that operation, and the follow-up neck CT revealed several lymph nodes (1–1.5 cm), some with cystic changes, over the posterior triangle of the left side of the neck. Excisional biopsy of the left supraclavicular lymph nodes yielded metastatic papillary thyroid carcinoma. He underwent total thyroidectomy and radical neck dissection 2 months later.

The pathology showed papillary microcarcinoma with characteristic features of nuclear crowding, optic clear and ground glass appearance and nuclear membrane irregularity with grooving (Figs. 2-1 and 2-2); the lesions were 0.3 and 0.1 cm in size and located in right and left lobes of the thyroid, respectively, with left supraclavicular lymph node metastasis (one of 5 lymph nodes was positive) and final stage pT1aN1Mx. After thyroidectomy and iodine-131 therapy, the patient was followed regularly. There was no recurrence or metastasis of the thyroid cancer.

At this visit, 18 years after previous concurrent adrenal GN and papillary thyroid carcinoma, the patient asked for a health examination and an evaluation of his prostate status. The digital rectal examination found no firm nodules, the PSA level was 1.587 ng/mL, and trans-rectal ultrasound showed a 46 mL prostate with no hypoechoic lesions.

**Fig. 3:**
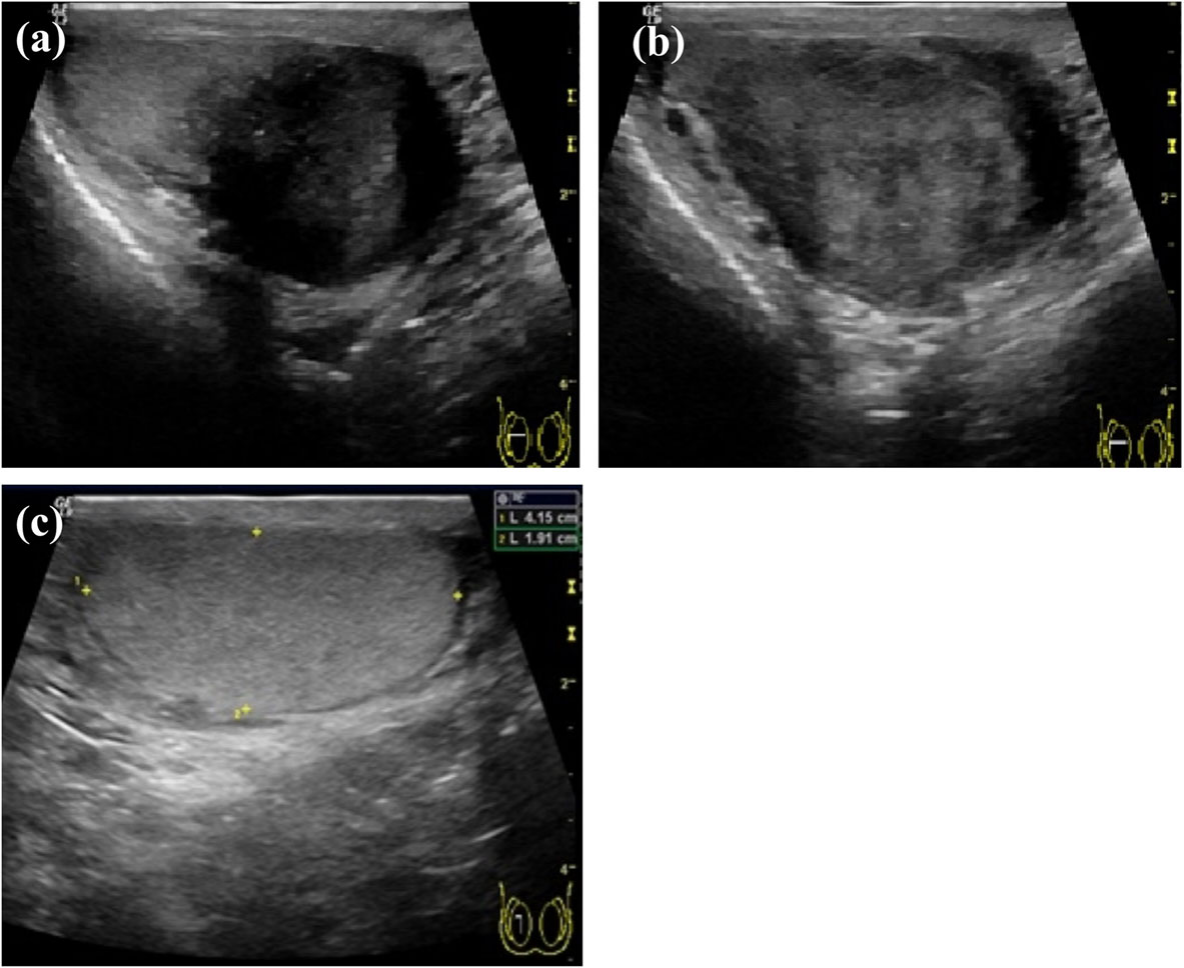
**a**-**c**. Para-testicular hypoechoic nodule over the right hemi-scrotum approximately 4.4 × 3.3 cm in size

The patient also complained of progressive enlargement of his right hemi-scrotum within the past 3 years. He had noted asymmetric sizes of the testes with a more prominent right hemi-scrotum since he was a teenager. Scrotal sonography revealed a 4.4 × 3.3 cm para-testicular hypoechoic nodule over the right hemi-scrotum (Fig. 3). The tumour was attached firmly to the right testis and appeared to originate from the epididymis. The laboratory tests for AFP, beta-human chorionic gonadotropin (β-HCG), and LDH yielded results of < 3.0 ng/ml, < 1.20 mIU/mL, and 156 IU/L, respectively.

**Fig. 4:**
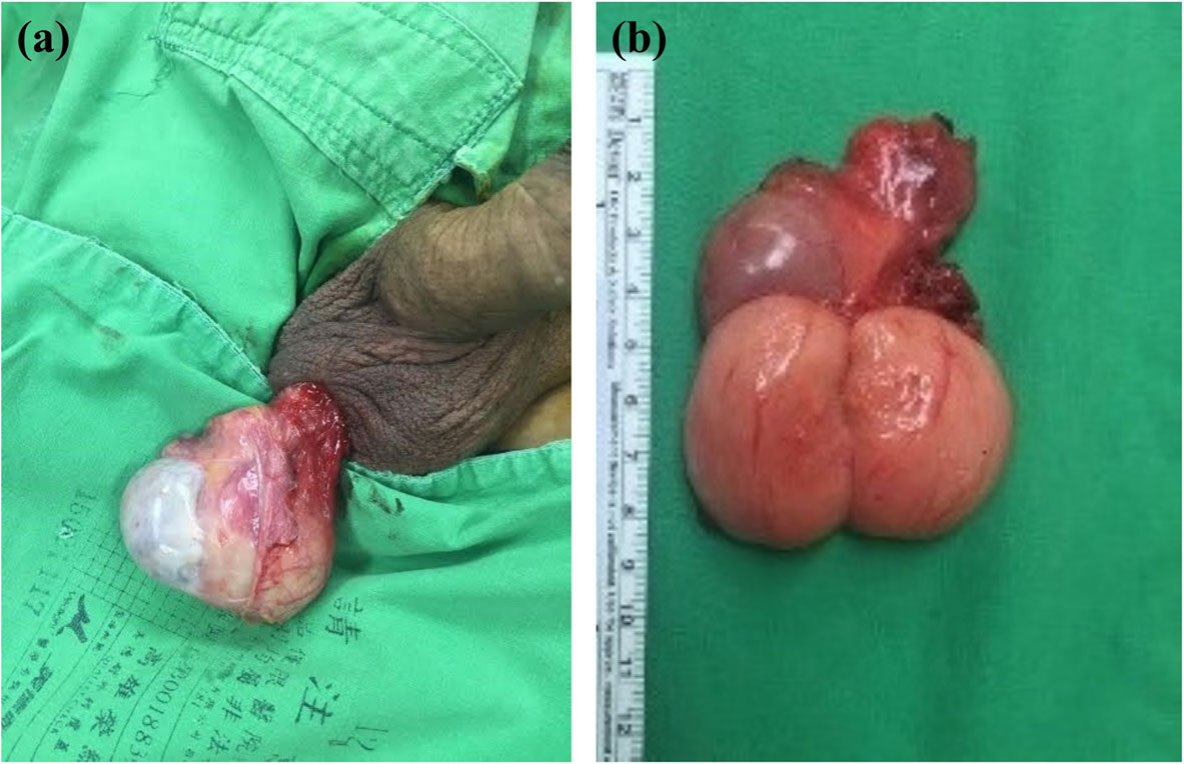
**a**. Paratesticular ganglioneuroma adhered closely to the testicular tunica albuginea. The tumour and part of the tunica albuginea are covered with a visceral layer of the tunica vaginalis. **b**. Bulging cut surface of the ganglioneuroma (lower) and testis (upper)

Under the impression of para-testicular tumor, the trans-scrotal orchiectomy was performed. Exploration of the right testis showed that the mass seemed to arise from the epididymis and was adhered firmly to the testis (Figs. 4-1 and 4-2). Operation was procceded following the standard procedure of inguinal orchiectomy, and this patient was discharged uneventfully 3 days after the surgery.

**Fig. 5:**
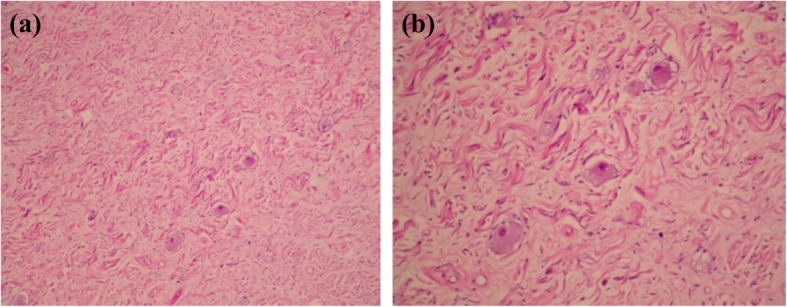
**a**. Paratesticular ganglioneuroma showing a neurofibromatous background decorated with dispersed mature ganglion cells. (HE, × 10). **b**. Higher magnification view of the paratesticular ganglioneuroma. (HE, × 20)

The pathology diagnosis was a paratesticular GN composed of a neurofibroma-like stroma decorated with scattered individuals and clusters of mature large ganglion cells containing an abundant eosinophilic cytoplasm and a prominent nucleolus. Primitive neuroblasts were absent. The parenchyma and epididymis of the testis were unremarkable. Microscopic pictures of the para-testicular GN over the right hemi-scrotum are shown in Figs. 5-1 and 5-2.

He then received regular follow-up, and post-operative chest tomography and thyroid sonography showed no signs of malignancy. However, a 0.8 cm globular optic nerve meningioma was discovered incidentally by whole-body MRI. Since there was no visual impairment or proptosis, the ophthalmologist and neurologist recommended regular clinical and radiological follow-up visits every 6 months.

## Discussion and conclusion

GN is usually observed in 10 to 30-year olds [12], and several studies have shown that it may also be observed between the ages 40 and 50 [13, 14]. Commonly reported locations include the posterior mediastinum, retroperitoneum, small intestine, colon and urinary bladder [4–7], while adrenal and para-testicular GN locations are very rare and are often unexpectedly discovered [9]. GN is usually silent and asymptomatic and becomes symptomatic due to hormone production or adjacent organ compression.

The majority of adrenal GNs manifest as adrenal incidentalomas, and a correct diagnosis is difficult to obtain preoperatively. It has been reported that more than 85% of adrenal incidentalomas are benign nonfunctioning adenomas [15], whereas some have hormone manifestations such as Cushing’s syndrome or pheochromocytoma. Thus, hormonal screening tests should be assessed to exclude functional tumours by means of the evaluation of urinary free cortisol and late-night salivary cortisol, the 1-mg overnight dexamethasone suppression test for Cushing syndrome, and the assessment of urinary catecholamines/metanephrines for pheochromocytoma [15].

Our patient, a 60-year-old man, experienced asymmetric sizes of the bilateral testes since he was a teenager. Sonography revealed a 4.4 × 3.3 cm para-testicular hypoechoic nodule in the right hemi-scrotum. His clinical course exemplifies the slow and indolent growth features of benign paratesticular GN [8]. It has been estimated that approximately 70% of paratesticular masses are benign, and the most common benign tumours are lipomas, adenomatoid tumours and leiomyomas [10, 11]. For the restremaining 30% of malignant paratesticular tumours, radical orchiectomy is recommended due to the high local recurrence rate. Intraoperative frozen section diagnosis can help with decision-making between focal resection and radical surgery. The tumour in our patient was noted to grow in size and was adhered firmly and thus could not be separated from the right testis during the operation. Therefore, we performed radical orchiectomy due to difficulty preserving the testis and concern about the possibility of malignancy.

There have been reports of spontaneous malignant transformation and malignant peripheral nerve sheath tumours arising from GNs [16, 17], as well as adrenal ganglioneuroma with hepatic metastasis [18], indicating the malignant potentials of GN. Therefore, close monitoring and possible surgery are needed.

To the best of our knowledge, this is the first case report of the metachronous occurrence of adrenal and paratesticular GN in the same patient. Whether there is any association between GN arising from the adrenal gland and from the paratesticular nerve system requires further discussion. However, this patient received whole-body MRI, and another tumour over the left orbital cavity with encasement of the left optic nerve was found during follow-up. He did not have related symptoms and considered surgery or biopsy for the tumour. It would not surprise us if the pathologic examination had also shown GN. There is no formal recommended surveillance for such patients. As these tumours are benign in nature, clinical monitoring is reasonable, but clinicians could consider whole body imaging (though it is not clear what to do if lesions are found).

In addition to that of paratesticular GN, the presentation of papillary thyroid carcinoma in our patient may indicate the association between GN and multiple endocrine neoplasms (MENs) [19, 20]. The recognized mechanism of MENs is attributed to mutation of the RET proto-oncogene, an oncogene encoding a tyrosine kinase receptor that is crucial for signal transduction in neural crest-derived tissues, which may include thyroid C cells, the enteric and peripheral nervous systems, and the chromaffin cells in the adrenal gland [21]. Thus, papillary thyroid carcinoma, pheochromocytomas and GN may originate from analogous neural crest cells. A clinical case report and study showed the manifestation of papillary thyroid carcinoma in MEN type 2 [22, 23] and RET/PCT rearrangement in papillary thyroid carcinoma [24, 25].

Furthermore, Lora MS, et al. reported adrenal ganglioneuromas in two paediatric patients with MEN type 2A and MEN type 2B. This finding suggests GN as a rare but not unexpected component of MEN2 syndromes [26]. Genetic testing, either of the tumours or of the germline, is crucial for the explanation of the presumed correlation. However, the test was not available in our lab, and the cost was prohibitive of referring testing. Thus, the patient did not want this test.

We reported the first patient with concurrent adrenal GN and papillary thyroid carcinoma who developed paratesticular GN eighteen years later. We assumed that there was an association among papillary thyroid carcinoma, GN and MEN2 syndromes. This case report may provide important information for the proposed association. However, further studies are required.

## Data Availability

Please contact author for data requests.

## Abbreviations

AFP: Alpha-fetoprotein
CEA: Carcinoembryonic antigen
CT: Computed Tomography
DHEAS: Dehydroepiandrosterone sulfate
GN: Ganglioneuroma
HCG: Human chorionic gonadotropin
LDH: Lactate Dehydrogenase
MENs: Multiple endocrine neoplasms
MRI: Magnetic Resonance Imaging
OPD: Out-Patient department
PCT: Papillary thyroid carcinoma
RET: REarranged during Transfection
VMA: Vanillylmandelic acid
